# Improved serological testing for bovine schistosomiasis in Eastern Africa

**DOI:** 10.1186/s13071-026-07332-1

**Published:** 2026-03-23

**Authors:** Veronika Tóth, Thomas A. Gasan, Bethany Crooks, Jahashi Nzalawahe, Alexandra Juhász, James E. LaCourse, J. Russell Stothard, Shaali M. Ame, Wei Hu, Geoffrey N. Gobert

**Affiliations:** 1 School of Biological Sciences, Queen’s University Belfast, Belfast, UK; 2 Sokoine University of Agriculture, Morogoro, Tanzania; 3 Department of Tropical Disease Biology, Liverpool School of Tropical Medicine, Liverpool, L3 5QA UK; 4 Institute of Medical Microbiology, Semmelweis University, Budapest, 1089 Hungary; 5 Public Health Laboratory, Ivo de Carneri, Pemba, United Republic of Tanzania; 6 Department of Infectious and Tropical Diseases, London School of Hygiene and Tropical Medicine, London, UK; 7 National Institute of Parasitic Diseases, Chinese Centre for Disease Control and Prevention (Chinese Centre for Tropical Diseases Research), National Health Commission Key Laboratory of Parasite and Vector Biology, WHO Collaborating Centre for Tropical Diseases, National Centre for International Research on Tropical Diseases, Shanghai, China; 8 The Institutes of Biomedical Sciences, College of Life Sciences, Inner Mongolia University, Hohhot, Inner Mongolia China

**Keywords:** Schistosomiasis, ELISA, Bovine, Diagnostic, Africa, *Schistosoma bovis*, *Schistosoma*, Serum, Cattle, One Health

## Abstract

**Background:**

In East Africa, bovine schistosomiasis, although common, is poorly appreciated and managed, detrimentally impacting upon livestock health. In certain settings, bovine schistosomiasis may be involved in zoonotic transmission of human schistosomiasis. Better disease management and more effective control of bovine schistosomiasis require the development of sensitive and specific serological screening and rapid diagnostic tools.

**Methods:**

We developed an enzyme-linked immunosorbent assay (ELISA) for *Schistosoma bovis* detection in cattle, utilizing nine shortlisted potential diagnostic protein targets. These shortlisted candidates, STI, IPP, OP, PGK1, COG, PDZ, and Sbp80 (as three fragments), were identified from *Schistosoma japonicum* homologs already reported with the highest diagnostic potential. In *S. bovis,* these proteins participate in various biological processes, including metabolic pathways, transcriptional regulation, glycolysis, phosphorylation, and cell signalling, although their real diagnostic potential has not been explored until now.

**Results:**

The ELISA was optimized using bovine blood serum samples from regions in Tanzania and validated for sensitivity and specificity. Two targets of specific focus, Conserved Oligomerix Golgi complex subunit 4 (COG) and a domain of the cysteine protease calpain (Sbp80), achieved the highest specificity and sensitivity among the recombinant proteins, with 92% and 88% sensitivity and 100% and 80% specificity, respectively. We further evaluated the COG-based and calpain-based ELISA on further “real-world” bovine serum samples from abattoir sites in Zanzibar, detecting *S. bovis* in 59.1% of tested animals.

**Conclusions:**

Both COG and calpain are promising candidates for serological screening and later inclusion in portable diagnostic tests for *S. bovis* infection in cattle. Such future diagnostic assays will enable better point-of-detection monitoring, and once scalable, aid in the control of disease in cattle.

**Graphical Abstract:**

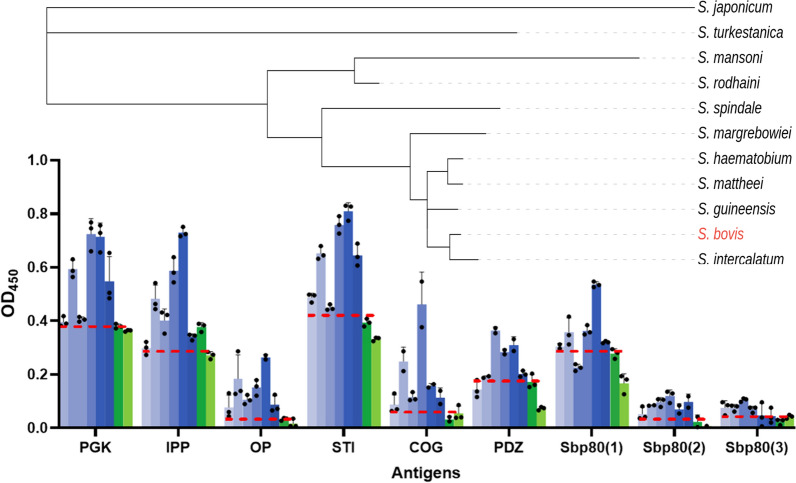

**Supplementary Information:**

The online version contains supplementary material available at 10.1186/s13071-026-07332-1.

## Background

Schistosomiasis is a neglected tropical disease (NTD), with both humans and animals at risk of infection by blood flukes of the *Schistosoma* genus [1, 2]. Key human species included *Schistosoma haematobium* and *S. mansoni* in Africa and *S. japonicum* in Asia. Infection of African livestock, such as cattle, includes *S. bovis*, *S. curassoni*, or *S. mattheei,* which are estimated to infect more than 165 million cattle worldwide [3], often resulting in enteritis, anemia, emaciation, and potentially, death of the animal [4]. The resulting economic impact of agricultural schistosomiasis is underappreciated, but often affects the poorest communities, already burdened by human schistosomiasis [5].

African schistosomes are transmitted through cercarial skin penetration to their definitive mammalian hosts via intermediate freshwater snails, with *Bulinus* being the key genus across the continent and adjacent regions. Other major genera, including *Biomphalaria* and *Oncomelania,* are also present in Africa or other parts of the world. After penetrating the epidermal barrier of the definitive mammalian host, they undergo complex maturation, resulting in diecious adults. Residing in the portal, mesenteric, and intestinal submucosal and subserosal vasculature, these adults produce eggs, which depending on the species, are excreted in either the feces or urine. Upon contact with fresh water, the eggs hatch to perpetuate disease transmission [6]. While some species of schistosome show specificity with their choice of mammalian host, others are more promiscuous. *Schistosoma bovis* is common in beef and dairy cattle [7, 8], but is also able to infect sheep, goats, and is now known to be zoonotic to humans, particularly in hybrid or introgressed forms [9]. Other schistosome species prevalent in cattle in Africa include *S. mattheei* and *S. curassoni* [10].

A combination of factors highlights agricultural hosts as a potential disease reservoir. These include the emergence of zoonoses and hybridization potential between schistosome species [11], as well as the relatively long (*c*. 7 weeks) maturation time [12]. No vaccine for any species causing schistosomiasis is currently available, and currently only one chemotherapy treatment is available: praziquantel, the production of which is almost exclusively for human medical use. This situation underlines the importance of ensuring that praziquantel continues to be effective in treating schistosome infections.

Diagnostic techniques for bovine schistosomiasis remain problematic, with technologies lagging behind those used for human schistosomiasis, and typically relying upon autopsies [13]. Direct parasitological methods, based on observation of eggs in cattle feces, include the miracidium hatching test (MHT) [14], the Danish Bilharziasis Laboratory technique [15], and formalin-ethyl acetate sedimentation digestion [15, 16]; while indirect molecular methods to detect *Schistosoma* DNA, such as quantitative polymerase chain reaction (qPCR) [17], are being used mainly for research purposes. However, these methodologies have known limitations such as low sensitivity at decreased infection intensities (MHT) [18, 19], challenges in obtaining fecal samples, and possible egg loss during prolonged sedimentation. The need for well-trained personnel, advanced laboratory equipment, and the often time-consuming nature of the processes involved can also make these techniques inapplicable in the field.

To improve the control and management of bovine schistosomiasis, user-friendly and effective serological diagnostic tests are critically needed. To address this, we utilized a framework of technologies originally developed for the detection of *S. japonicum* in humans [20] to enhance the detection of *S. bovis* in cattle for application in Africa.

## Methods

### Identification of targets

*Schistosoma bovis* diagnostic targets were identified using an in silico approach (Table 1). The top-performing established diagnostic proteins from *S. japonicum* were identified using the area under the receiver operating characteristic (ROC) curve (AUC) and a two-tailed unpaired Student’s *t*-test (as described by Chen and colleagues [20]). Using the tBLASTn function of WormBase ParaSite (https://parasite.wormbase.org), the *S. japonicum* protein sequences were used as a query sequence to interrogate the *S. bovis* genome (PRJNA451066, version GCA_003958945.1) [21]. Successful homolog hits were deemed to have an expected threshold of less than 1.0 × 10^−5^. Additionally, iterative searches were conducted on the closely related [22] *S. haematobium* to help infer the full-length *S. bovis* CDS of any genes identified.

**Table 1:** *Schistosoma bovis* homologs of top-performing *S. japonicum* diagnostic targets and calpain fragment design

Name	*S. japonicum* homolog	AUC	*S. bovis* Gene ID	Description	eValue
STI	SJCHGC06661	0.95	DC041_0008768	STIP1 and U-box containing protein 1	1.90E-06
IPP	SJCHGC07024	0.93	DC041_0005880	Inorganic pyrophosphatase/diphosphatase	1.90E-54
OP	SJCHGC05998	0.91	DC041_0002182	Oligo peptidase/ubiquitin thioesterase	7.30E-73
PGK1	SJCHGC09293	0.9	DC041_0012484	Phosphoglycerate kinase	4.40E-163
COG	SJCHGC04941	0.9	DC041_0005454	Conserved Oligomeric Golgi complex, subunit 4	4.20E-144
PDZ	SJCHGC04616	0.9	DC041_0005385	PDZ containing protein	2.10E-109

*S. bovis* calpain domains DC041_0009470			
Fragment	Fragment start (bp)	End bp	Size bp
Sbp80(1)	0	320	320
Sbp80(2)	150	450	300
Sbp80(3)	300	678	378

In addition to the in-silico-derived targets, the calcium-dependent cysteine proteases, calpain (Sbp80), were included [23] as a supplementary antigen. Predominantly expressed on the tegument, calpain is an intensively studied vaccine target for *S. mansoni* [24] and was recently characterized in *S. mekongi* [25] (Table 1).

### Plasmid construction

After the addition of *Alw*N and *Sbf*I restriction sites to the 5’ and 3’ ends, respectively, finalized expression sequences were codon-optimized for expression in *Escherichia coli* and submitted for gene synthesis by GeneArt (ThermoFisher, Regensberg, Germany). Due to the large size of calpain (Gene ID: DC041_0009470), it was divided into three overlapping peptide fragments: Sbp80(1), Sbp80(2), and Sbp80(3), measuring 320 bp, 300 bp, and 378 bp, respectively, to ensure full gene coverage. Gene inserts were separated from the provided pMX shuttle vector by double restriction digest and ligated into the *Alw*N/*Sbf*I sites of the pMALc6-T expression vector. To ensure the correct reading frame and insert gene, the pMAL constructs were sent for DNA sequencing to Eurofins Genomics (Cologne, Germany).

### Recombinant protein production

Plasmid constructs were transformed into NEBExpress *E. coli* (New England Biolabs, Ipswich, USA) as per the manufacturer’s instructions, and used to inoculate 1 L of culture media (10 g tryptone, 5 g yeast extract, 5 g NaCl, and 2 g glucose, with 100 µg/ml of ampicillin) (Merck, Darmstadt, Germany). At Optical Density OD = 0.6, the cultures were induced with a final concentration of 0.3 mM isopropyl β-D-1-thiogalactopyranoside (IPTG, Melford, Ipswich, UK) and protein expression was allowed to continue for 4 h at 30 °C. After incubation, the cells were harvested by centrifugation (4000 × *g*, 20 min), resuspended in column buffer (CB; 20 mM Tris–HCl, 200 mM NaCl, 1 mM ethylenediaminetetraacetic acid (EDTA), 1 mM DL-dithiothreitol (DTT), pH 7.5; Merck, Darmstadt, Germany) containing Complete Protease Inhibitor Cocktail (Roche, Basal, Switzerland), and lysed by sonication (12 × 15 s, with 10 s intervals on ice; GT SONIC, Ultrasonic Cleaner VGT-1620Q TD, Meizhou City, China). The resulting soluble and insoluble fractions were separated by centrifugation at 21,000 × *g* for 20 min.

Purification of the soluble protein was achieved using Amylose resin (New England Biolabs, Ipswich, USA) and eluted with CB containing 10 mM maltose (Merck, Darmstadt, Germany) according to the manufacturer’s instructions.

### Biological sample collection and processing

Cattle were sampled in two distinct periods to serve different purposes. All Tanzanian cattle were sampled first to support ELISA assay development, while all Zanzibar cattle were sampled subsequently for assay validation. Sampling was opportunistic (convenience-based) at multiple sites, including official government abattoirs and farms, with several animals sampled per site.

Biological specimens (adult *Schistosoma* worms, blood, stool) were collected from cattle at multiple sites in Tanzania (Iringa and Arumeru District councils of the Iringa and Arusha regions) and Zanzibar (Muwanda), following locally established protocols. All specimens were processed by trained local laboratory teams. Preserved male and female worms and frozen serum samples were shipped on ice according to transfer regulations to Queen’s University Belfast (QUB), and upon arrival, stored at −80 °C or short term at −20 °C until needed.

#### Miracidial hatching test

Collected stool samples were tested for live miracidia by the hatching method: in Tanzania, the MHT was performed on the stool samples a day before the slaughter, as per Liang and colleagues [26]. In Zanzibar, the stool samples were checked for miracidia as previously described [14, 18]. Briefly, each sample (approximately 15 g) was homogenized and sieved through successive layers of mesh (400, 180, 30 µm) to remove plant/other debris and isolate eggs. The final sediment was placed in a Petri dish filled with freshwater (tap water; pH around 7.0) that had been left to stand for ≥ 24 h to allow time for dechlorination. Water used for hatching assays was free from visible debris and never taken from sites with potential schistosome egg contamination. The samples were exposed to artificial or natural light at room temperature (RT, ranging from 25 °C to 30 °C) for 10 min to allow miracidia to hatch. Afterward, the samples were examined under a stereomicroscope for approximately 4 min to detect the presence of miracidia. If no miracidium could be detected during this time, the sample was scored as negative for infection.

#### Collection of *Schistosoma* worms

Adult *Schistosoma* worms were collected from Tanzanian cattle (an indigenous breed) that were naturally infected with *Schistosoma* worms (infection confirmed by the MHT described in Miracidial hatching test). Multiple adult male and female worms were collected from the mesenteric veins of each infected animal during postmortem examination immediately following slaughter in the abattoirs. The worms were kept separately per individual host throughout processing. Worms were washed three times in phosphate-buffered saline (PBS; Sigma-Aldrich, St. Louis, USA) to remove host material, and stored in RNA*later* Solution (Thermo Fisher Scientific, Waltham, USA) or 70% ethanol. For downstream molecular analyses, only adult male worms were used for species identification.

#### Blood sample collection

Blood samples were collected from a total of 79 cattle in Tanzania (*n* = 35) and Zanzibar (*n* = 44). In Tanzania, 25 animals were infected with *S. bovis* (“true positives,” infection confirmed by the MHT as described in Miracidial hatching test), with 10 animals uninfected (“true negatives”). All uninfected animals originated from the regions of Iringa and Arumeru District councils, where no *S. bovis* infections have been reported. Blood was collected following the methods outlined in [27] with some modification: 35 ml of blood was collected from each animal at slaughter. Blood samples were left to coagulate at RT for up to 2 h and refrigerated overnight at 4 °C. Samples were centrifuged at 10,000 × g for 10 min to separate serum from erythrocytes. In the absence of a centrifuge, blood was kept at 4 °C until complete retraction of the clot from the serum (no longer than 24 h). Serum (10 ml) was pipetted off and stored at −20 °C or at −80 °C. In Zanzibar, blood samples were collected from field sites and abattoirs in Muwanda using an opportunistic approach. Blood samples were obtained from live cattle by jugular venipuncture using a sterile syringe before slaughter. Approximately 2 ml of blood was collected from each animal into an EDTA tube and centrifuged at 4000 × *g* for 5 min. Serum was harvested into a new 2 ml tube and snap frozen at −20 °C.

### Speciation of adult schistosome worms from mainland Tanzania

Adult *Schistosoma* worms were kept at −20 °C in RNA*later *Solution (Thermo Fisher Scientific, Waltham, USA) prior to genomic DNA (gDNA) extraction. Samples were thawed on ice and individual male worms were transferred into RNase-free tubes (1 worm/tube). Worms were snap frozen in liquid nitrogen before the addition of a single clean, sterile 5-mm stainless steel bead (Qiagen, Hilden, Germany). Worm tissue was homogenized by bead beating at 50 Hz for 4 min using the TissueLyser LT (Qiagen, Hilden, Germany). After bead beating, the lysate was transferred into a new RNase-free tube, and 180 µl of tissue lysis buffer (Buffer ATL, Qiagen, Hilden, Germany) was added. gDNA was extracted using the DNeasy® Blood and Tissue kit (Qiagen, Hilden, Germany) according to the manufacturer’s instructions with the 4 h Proteinase K (Qiagen, Hilden, Germany) incubation at 56 °C. The quantity and quality of the extracted gDNA were checked using Denovix Nanodrop (Thermo Fischer Scientific, Waltham, USA). Typically, between 5 and 30 ng of gDNA was extracted from a single adult *Schistosoma* worm in 80 µl of the buffer AE (Qiagen, Hilden, Germany). Extracted gDNA was stored at −20 °C.

The conventional PCR method was used to speciate *Schistosoma* worms collected from Tanzanian cattle. Previously reported primers targeting the nuclear ribosomal internal transcribed spacer (rITS) gene of schistosomes [9, 28] were used and generated by Eurofins Genomics (Cologne, Germany). Primer sequences were as follows: forward rITS primer 5’ TAACAAGGTTTCCGTAGGTGAA ‘3; reverse rITS primer 5’ TGCTTAAGTTCAGCGGGT ‘3. Taq PCR mastermix kit (Qiagen, Hilden, Germany) was used to amplify the rITS gene. Reaction volume was 50 µl, consisting of 5 µl of template DNA, 26 µl of Taq PCR Master Mix, 2 µl of 100 µM forward and reverse primer (final concentration 4 µM of each primer), and 16 µl of DEPC-treated water. For the negative control, 5 µl of DEPC-treated water was used instead of the DNA template. We used gDNA from adult male and female *S. mansoni,* Strain NMRI (NR-28910, BEI Resources, Manassas, USA) as a positive control in these experiments. The thermocycler (MiniAmp, Applied Biosystems, Waltham, USA) was programmed to 10 min of initial denaturation at 95 °C, which was followed by 35 cycles of 95 °C for 15 s, 56 °C for 15 s, and 72 °C for 1 min, and a final extension for 10 min at 72 °C. The size of the amplified products (expected amplicon size of approximately 1005 bp) was checked on 1% agarose gel that was run at a constant 110 V for 40 min (NuPAGE, Thermo Fisher Scientific, Waltham, USA). Amplified DNA was purified using the Gel Clean-Up Kit (Qiagen, Hilden, Germany) and sent for sequencing (TubeSeq service, Eurofins Genomics).

The obtained rITS sequences from TubeSeq sequencing service were aligned, and variable sites were analyzed using Basic Local Alignment Search Tool (BLAST, [29]) against all known *Schistosoma* species in the NCBI nucleotide database and WormBase ParaSite (https://parasite.wormbase.org). For each sequence, the top BLAST hit, accession number, E-value, percent identity, and query coverage were recorded (Supplementary Table 1). On the basis of high similarity (≥ 99.68%) observed among the 24 full-length rITS sequences, 1 representative sequence (969 bp) was selected for GenBank submission. In addition, a partial rITS sequence (77 bp) obtained from an additional worm was also submitted.

### Enzyme-linked immunosorbent assay (ELISA)

Prior to experiments with target antigens, the antigenicity of both positive and negative blood sera from Tanzania and Zanzibar was checked using ELISA (described below) coated with 2 µg/well of lipopolysaccharides (LPS) from *Escherichia coli* O111:B4 (LPS; Sigma, Gillingham, United Kingdom). This was done to ensure the integrity of the antibodies had not been compromised during transport.

The ELISA was performed as follows: high-binding 96-well plates (Immulon® 2 HB, Thermo Fisher Scientific, Waltham, USA) were coated with recombinant *S. bovis* PGK, IPP, OP, STI, COG, PDZ, Sbp80(1), Sbp80(2), Sbp80(3) antigens (or the LPS), 2 µg/well diluted in 1X PBS (50 µl/well; MilliporeSigma, Burlington, USA) in triplicate, and incubated overnight at 4 ℃. Additionally, each plate contained the no-antigen-no serum control consisting of 50 µl/well of 1X PBS. The serum samples collected from *S. bovis*-infected and uninfected cattle were thawed on ice. The Immulon 2 HB 96-well Flat Bottom MicroTiter plates (Thermo Fisher Scientific, Waltham, USA) were blocked with 200 µl/well of 5% milk (Marvel, United Kingdom) diluted in 1X PBS containing 0.05% TWEEN® 20 (Sigma-Aldrich, St. Louis, USA; PBS-T-Milk 5%), and incubated for 2 h at RT, before washing six times with PBS-T (1X PBS containing 0.05% TWEEN® 20). Next, 50 µl/well of individual bovine blood serum samples diluted 1:25 in PBS-T supplemented with 1% bovine serum albumin (BSA; Sigma-Aldrich, St. Louis, USA; PBS-T-BSA 1%) were added in triplicate into the wells, before plates were incubated for 90 min at 37 ℃. In pooling experiments, the serum master mix was prepared from serum from four individual animals and diluted 1:50 with 1X PBS. For the no-antigen-no-serum control wells, 50 µl/well PBS-T-BSA 1% was used, as well as the no-serum control wells (used to measure the baseline absorbance). Plates were again washed six times with PBS-T and incubated with 50 µl/well of secondary goat anti-bovine IgG HRP antibody (Invitrogen, Carlsbad, USA), diluted 1:10,000 in PBS-T-BSA 1%, for 90 min at 37℃. Following incubation, the plates were washed six additional times with PBS-T. The antigen–antibody interaction was then developed by adding 100 µl/well of 3,3′,5,5′-Tetramethylbenzidine (TMB) solution (Sigma-Aldrich, St. Louis, USA), prepared according to the manufacturer’s instructions. Plates were incubated for 25 min in the dark, and the enzymatic reaction was stopped with the addition of 100 µl/well of 1 M sulfuric acid (Honeywell™, Morris Plains, USA). The signal was read at 450 nm using FLUOstar Omega Microplate Reader (BMG Labtech, Ortenberg, Germany), and samples were analyzed using an absorbance threshold defined by the ROC curve analysis. Samples with absorbance values above the threshold were considered positive in the diagnosis of infection.

### Statistical analysis

Statistical analyses were conducted using GraphPad Prism, version 8.0 (San Diego, CA, USA). First, the baseline absorbance (average of the absorbance value measured in the no serum control wells) was subtracted from the absorbance values measured from the wells that contained bovine serum samples to correct for background noise and obtain the net absorbance due to antigen–antibody binding. The normal distribution of the samples was evaluated using the Shapiro–Wilk normality test. The comparison of the mean absorbance readings between the positive and negative groups was analyzed using the Mann–Whitney test. The threshold values were determined by the ROC curve using 25 serum samples from cattle positive for bovine schistosomiasis and 10 serum samples that were obtained from cattle negative for bovine schistosomiasis, and the results (sensitivity and 1-specificity) were expressed as percentages. The 95% confidence intervals were calculated using the Wilson/Brown method. Positive samples were those with absorbance readings above the established ROC cutoff, while negative samples were those with absorbance readings below the cutoff. Similarly, threshold values for blood serum samples from Zanzibar were determined using an ROC curve using *S. bovis* positive (all) and *S. bovis* negative serum (obtained from the field sites and not the abattoir)—as identified by the MHT. Those samples in which miracidia hatched within 4 min were considered *S. bovis* positive. Negative and positive prediction values (NPV, PPV) were calculated as described previously [30]. The percentage difference in OD450nm was calculated using:

$$\frac{av. positive-av. negative }{av. negative}\times 100$$

## Results

### Molecular analysis of *Schistosoma* specimens

Species identification was performed on 25 individual adult male worms, each collected from one of the 25 Tanzanian indigenous breed cows, and undertaken via amplification and BLAST analysis [29] of the nuclear rITS region. This showed that worms from 24 of 25 cows matched *S. bovis* rITS (accession OX104095.1) with 99.7–100%. Accordingly, one representative rITS sequence was submitted to GenBank (accession PX854844), along with a second sequence consisting of a short rITS fragment (77 bp) obtained from an additional worm from the remaining cow (accession PX857471). Repeated sequencing attempts on worms from this cow were unable to generate a full-length amplicon. Nonetheless, BLAST analysis of the 77 bp fragment placed the sequence within the *Schistosoma* genus (90.79% identity), but its short length precluded species-level assignment. The main information for the top BLAST hits for each sequence (accession number, E-value, percent identity, and query coverage) is recorded in Supplementary Table 1. Collectively, these results indicate that the worms obtained from Tanzanian cattle were predominantly *S. bovis.*

### ELISA initial antigen screen

**Fig. 1:**
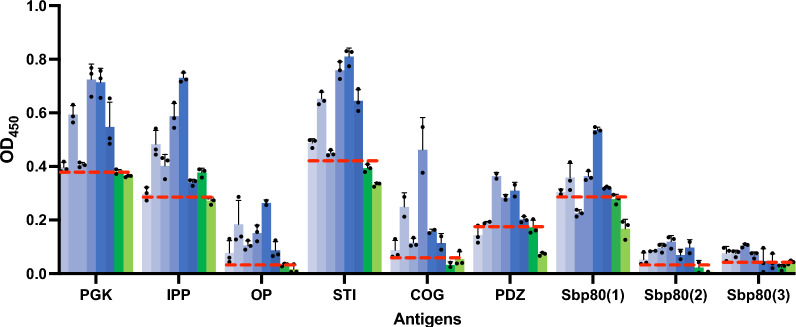
ELISA probing recombinant *S. bovis* tegument proteins with pooled Tanzanian cattle sera. Optical density at 450 nm is shown for animals with (“true positive,” blue bars) and without (“true negative,” green bars) *S. bovis* infection, determined by MHT. Positive and negative pooled samples were made from four individual cows per pool. The ROC cutoffs (thresholds), determined per antigen, are shown as red hashed lines. Samples with absorbance readings above the ROC cutoff are classed as positive, while those below the cutoff are negative

Initially, ELISA with pooled sera from Tanzanian cattle was used to screen nine shortlisted antigens that were recombinantly produced in our laboratory. Significant area under the curve (AUC; *P* = 0.045) in the ROC analysis of ELISA indicated that PGK, STI, COG, and Sbp80(2), and Sbp80(3) are effective in distinguishing between *S. bovis*-positive and *S. bovis*-negative animals. The ELISA performed using the abovementioned proposed diagnostic proteins showed 100% specificity and 100% sensitivity in pooled sera experiments (Table 2; Fig. 1). The ROC analysis of the ELISA performed with IPP, OP, PDZ, and Sbp80(1) did not yield statistically significant findings (*P* = 0.182, *P* > 0.999, *P* = 0.097, and *P* = 0.097, respectively), suggesting their limiting diagnostic potential (Fig. 1).

**Table 2:** ROC analysis of ELISA using recombinant *S. bovis* tegument proteins for detection of *S. bovis* infection

Antigen	Threshold	Sensitivity %	95% CI	Specificity %	95% CI	AUC	95% CI	%DOD
PGK	0.388	100.00	60.97%, 100.00%	100.00	17.77%, 100.00%	1.00	1.00%, 1.00%	51.6
IPP	0.285	100.00	60.97%, 100.00%	50.00	2.57%, 97.44%	0.83	0.51%. 1.00%	46
OP	0.034	100.00	60.97%, 100.00%	50.00	2.57%, 97.44%	0.50	0.00%, 1.00%	452
STI	0.422	100.00	60.97%, 100.00%	100.00	17.77%, 100.00%	1.00	1.00%, 1.00%	74.6
COG	0.071	100.00	60.97%, 100.00%	100.00	17.77%, 100.00%	1.00	1.00%, 1.00%	300
PDZ	0.182	83.33	43.65%, 99.15%	100.00	17.77%, 100.00%	0.92	0.70%, 1.00%	93.9
Sbp80(1)	0.291	83.33	43.65%, 99.15%	100.00	17.77%, 100.00%	0.92	0.70%, 1.00%	59.3
Sbp80(2)	0.034	100.00	60.97%, 100.00%	100.00	17.77%, 100.00%	1.00	1.00%, 1.00%	394
Sbp80(3)	0.044	100.00	60.97%, 100.00%	100.00	17.77%, 100.00%	1.00	1.00%, 1.00%	113

Thresholds, AUC levels, and %DOD_450_ were determined for each antigen. PGK, STI, COG, Sbp80(2), and Sbp80(3), reached sensitivity and specificity of 100% and proved effective in differentiating *S. bovis*-positive from *S. bovis*-negative samples with no false classifications

### Conserved Oligomerix Golgi complex subunit 4 (COG) identified as a main diagnostic target

**Fig. 2:**
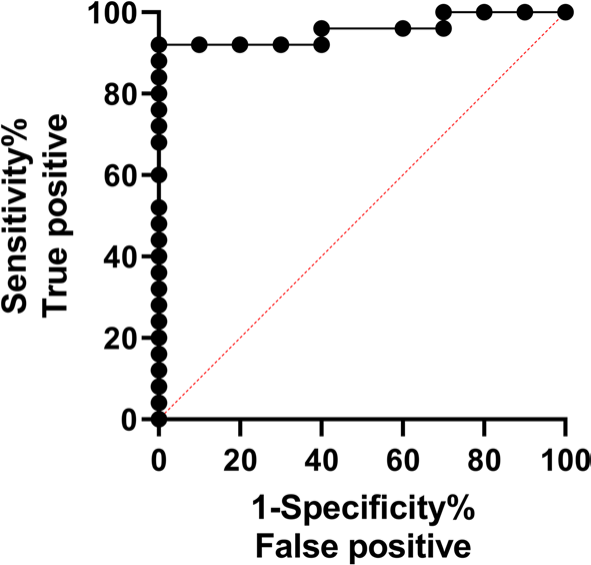
ROC curve of the leading diagnostic antigen COG using serum from mainland Tanzania

**Fig. 3:**
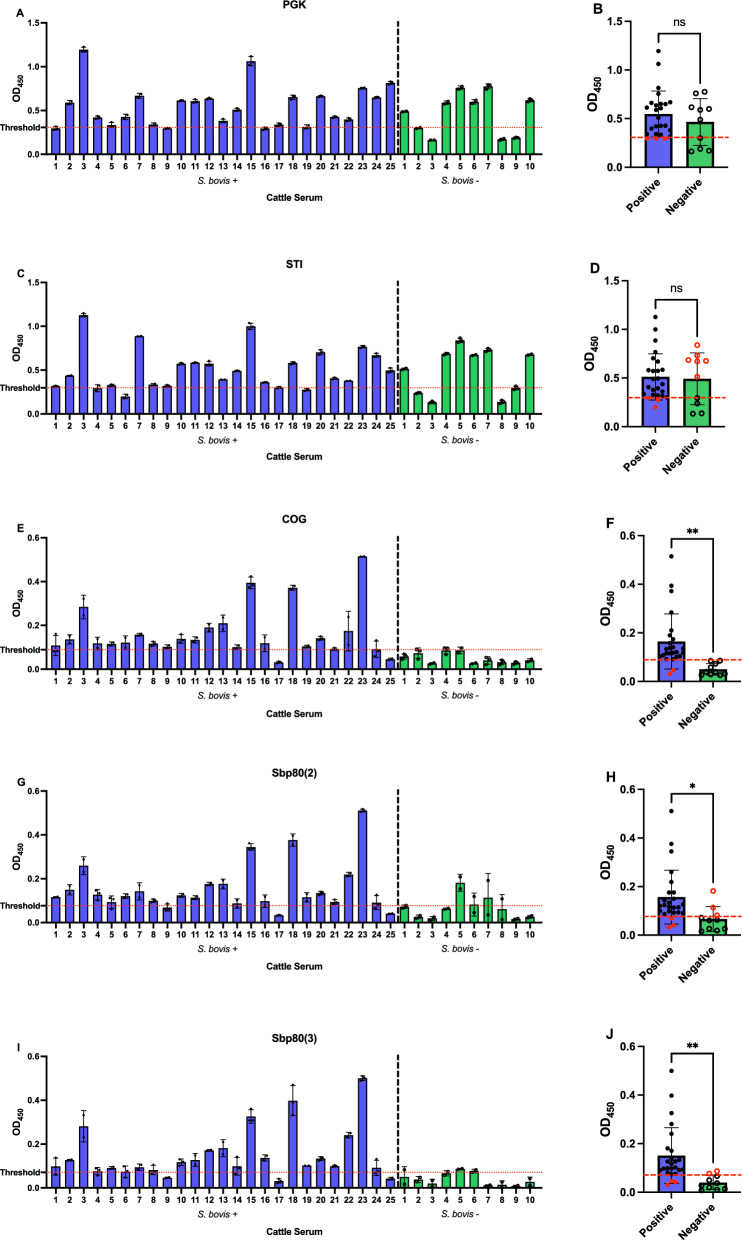
Performance of diagnostic proteins for detecting *S. bovis* by ELISA, with serum samples of mainland Tanzania cattle. **A**, **C**, **E**, **G**, **I** Optical density at 450 nm is shown for animals with (“true positive,” blue bars, *n* = 25) and without (“true negative,” green bars, *n* = 10) *S. bovis* infection, determined by MHT. Samples were classified as serologically positive or negative on the basis of the ROC cutoff value (dashed red line), determined per antigen. The AUC levels determined using the ROC curve analysis are shown in Fig. 2. **B**, **D**, **F**, **H**, **J** Comparison of ELISA absorbance values between “true positive” and “true negative” animals. Each point represents an individual animal, with false positives and false negatives in red (significance is shown as *P < 0.05, **P < 0.01)

ELISA was further optimized for the validation of the PGK, STI, COG, Sbp80(2), and Sbp80(3) as diagnostic proteins with the focus on sensitivity and specificity. The ELISAs conducted on the COG, Sbp80(2), and Sbp80(3) with individual samples of Tanzanian cattle serum revealed significant AUC in the ROC assessment (*P* < 0.001, *P* < 0.002, *P* < 0.001, respectively). COG demonstrated superior performance in the ELISAs, as determined by ROC analysis (Fig. 2), scoring the highest NPV and PPV, with scores of 1 and 0.92, respectively (Table 3). COG also exhibited a specificity of 100%, indicating its remarkable ability to accurately exclude all individuals without *S. bovis* infection. Additionally, COG showed to be 92% sensitive, highlighting its capacity to correctly identify 23 out of 25 *S. bovis*-infected cattle (Fig. 3E). These findings underscore the effectiveness of COG as a robust biomarker for novel *S. bovis* diagnostic tools. The specificity of Sbp80(2) and Sbp80(3) in ELISAs reached 70% and 80%, respectively, and 88% sensitivity (Table 3; Fig. 3G, I). Furthermore, COG, Sbp80(2), and Sbp80(3) showed a large difference in OD_450_ values between *S. bovis*-positive and *S. bovis*-negative animals, with 219.88%, 137.91%, and 284.58% higher average OD_450_ in the positive group, respectively (Table 3 and Fig. 3F, H, J).

**Table 3:** ROC analysis performed to determine the capability of each diagnostic antigen in ELISA to discriminate the *S. bovis*-negative (*n* = 10) and *S. bovis*-positive animals (*n* = 25)

Antigen	Threshold	Sensitivity (%)	95% CI	Specificity (%)	95% CI	AUC	95% CI	NPV	PPV	%DOD
PGK	0.307	88.00	70.04%, 95.83%	40.00	16.82%, 68.73%	0.5920	0.3624%, 0.8216%	0.4	0.88	16.25
STI	0.298	88.00	70.04%, 95.83%	40.00	16.82%, 68.73%	0.5120	0.2622%, 0.7618%	0.4	0.88	9.24
COG	0.089	92.00	75.03%, 98.58%	100.00	72.25%, 100.0%	0.9560	0.8898%, 1.000%	1.0	0.92	219.88
Sbp80(2)	0.077	88.00	70.04%, 95.83%	70.00	39.68%, 89.22%	0.8420	0.6785%, 1.000%	0.7	0.88	137.91
Sbp80(3)	0.071	88.00	70.04%, 95.83%	80.00	49.02%, 96.45%	0.9280	0.8457%, 1.000%	0.8	0.88	284.58

Thresholds, AUC levels, NPV, PPV, and %DOD_450_ were determined for each antigen

Results of the ROC analysis of the ELISA performed with the PGK and STI antigens revealed that their AUC values were not statistically significant (*P* = 0.401, *P* = 0.913, respectively), indicating poor discriminatory power between *S. bovis*-positive and *S. bovis*-negative serum samples on the individual level (Table 3; Fig. 3B, C). Although both PGK and STI demonstrated a sensitivity of 88% in correctly identifying *S. bovis*-positive animals, their specificity of 40% resulted in a high rate of false positives in our assays (Table 3; Fig. 3A, C).

### *S. bovis* detection in Zanzibar cattle samples

Following the successful laboratory validation of COG and Sbp80(3) as the top-performing *S. bovis* diagnostic proteins, we selected one additional location in East Africa, Zanzibar, to initiate pilot testing of the optimized ELISA with the “real-world” samples.

**Fig. 4:**
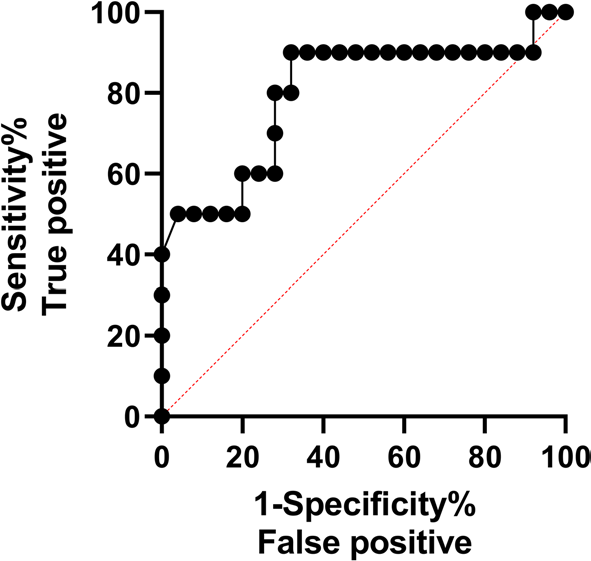
ROC curve of the leading diagnostic antigen, COG, for the diagnosis of *S. bovis* infection in tested cattle in Zanzibar using ELISA

**Fig. 5:**
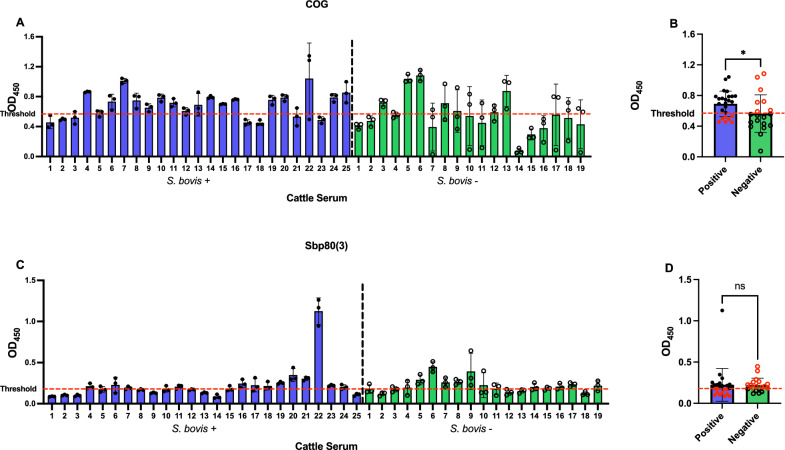
Performance of diagnostic proteins for detecting *S. bovis* by ELISA, with serum samples of Zanzibar cattle. **A**, **C** Optical density at 450 nm is shown for animals with (“true positive,” blue bars, *n* = 25) and without (“true negative,” green bars, *n* = 19) *S. bovis* infection, determined by MHT. Samples were classified as serologically positive or negative on the basis of the ROC cutoff value (dashed red line), determined per antigen. The AUC levels determined using the ROC curve analysis are shown in Fig. 4. **B**, **D** Comparison of ELISA absorbance values between the positive and negative animals (significance is shown as *P < 0.05). Each point represents an individual animal, with false positives and false negatives in red

The MHT was used to determine whether Zanzibar cattle in the field were infected with bovine schistosomiasis. This technique reported a 56.8% prevalence in tested animals (25 out of 44). Serum samples from both *S. bovis*-infected and non-infected cows were assessed individually against the leading target proteins using ELISA. COG demonstrated superior performance in ELISA compared with Sbp80(3), correctly identifying a higher proportion of positive cases (72% versus 56%), and was more dependable in identifying *S. bovis*-negative animals (63% versus 37%; Table 4; Figs. 4; 5). This is reflected in a PPV of 0.72 and NPV of 0.63. Among the sampled cattle population, *S. bovis* was detected in 59.1% (26 out of 44) of animals using COG, which showed greater diagnostic accuracy.

**Table 4:** ROC analysis performed to determine the optimal threshold for identification of the *S. bovis*-negative and *S. bovis*-positive animals

Antigen	Threshold	Sensitivity (%)	95% CI	Specificity (%)	95% CI	AUC	95% CI	NPV	PPV	%DOD
COG	0.568	80.00	49.02%, 96.45%	72.00	52.42%, 85.72%	0.798	0.6134%, 0.9826%	0.64	0.72	22.6
Sbp80(3)	0.179	70.00	39.68%, 89.22%	44.00	26.67%, 62.93%	0.504	0.3117%, 0.6963%	0.26	0.56	1.65

Thresholds, AUC levels, NPV, PPV, and %DOD_450_ were determined for COG and Sbp80(3) and are presented in the table

The ROC analysis of Sbp80(3)-based ELISA suggested a low level of diagnostic ability, with the AUC of 0.504. Although Sbp80(3) reached the sensitivity of 70%, the specificity of Sbp80(3) in this setting was relatively poor (44%), leading to a higher rate of false positives and reduced accuracy in distinguishing negative cases (Table 4; Fig. 5).

## Discussion

Control and elimination of schistosomiasis is listed as one of the current priorities in the NTD roadmap 2021–2030 of the World Health Organization (WHO) [31]. Central to accomplishing this goal is the ability to timely and accurately diagnose schistosome infections in both humans and animals, which allows for the effective administration of an appropriate treatment while minimizing selection pressure on worms to become praziquantel-resistant. Applicable diagnostics are also important for the determination of assigning, and subsequent monitoring, endemic areas receiving community-wide mass drug administration (MDA) to measure the impact of MDA in reducing intensity and prevalence of infections. These considerations are necessary for the mapping of schistosome transmission dynamics between humans and agricultural host reservoirs and to better consider the zoonotic nature of the worms and any emerging hybrid species [32].

The development of effective and field-friendly diagnostic tools for bovine schistosomiasis is an important task that needs to be addressed. We identified here two *S. bovis* proteins with high diagnostic potential and demonstrated the capacity of the antibody-based detection ELISA to detect *S. bovis* infections in cattle in East Africa, with elevated levels of both sensitivity and specificity. Animal schistosomiasis, particularly in livestock, affects an estimated 165 million cattle worldwide and has long-term impacts on growth, fertility, and disease susceptibility, leading to considerable economic losses [3]. Bovines may contribute to the transmission cycle of schistosomiasis given the zoonotic potential of schistosomes [9] and the emergence of hybrid species capable of infecting humans [11]. This role is especially concerning during outbreaks, as individual cattle can excrete daily between 10 and 1000 eggs per gram of feces into the environment [3]. Given that cattle can produce up to 25 kg of feces a day [33], they represent a significant source of disease transmission to the intermediate host snails wherever freshwater becomes contaminated. Therefore, addressing bovine schistosomiasis could play an important role in achieving the WHO’s 2030 elimination targets [31]. Lack of accurate diagnostic tools can result in the underestimation of disease prevalence and exposure, underscoring the need for more effective diagnostic approaches to combat schistosomiasis in affected regions.

Our ELISA, performed using blood serum samples from Tanzanian cattle, identified COG (subunit 4) and a domain of the cysteine protease calpain (Sbp80(3)) as the best-performing candidates for serological diagnosis of *S. bovis* infection. COG’s homolog from *S. japonicum* was determined as one of the 30 most highly immunoreactive and antigenic tegument proteins in *S. japonicum* [20], highlighting its potential as a diagnostic and druggable target. The COG protein complex is evolutionarily conserved and contains eight subunits that play a role in maintaining Golgi structure and function and in intra-Golgi trafficking [34, 35]. A BLAST search on COG’s subunit 4 revealed conservation across *Schistosoma* species (Supplementary Fig. 1, Supplementary Table 2), suggesting that it could serve as a reliable diagnostic target within the genus. This conservation may be particularly advantageous in regions where multiple *Schistosoma* species are co-endemic, enabling broad diagnostic coverage with a single test. However, its conservation beyond the genus *Schistosoma* and into other parasitic species within the family Schistosomatidae raises concerns about potential cross-reactivity. This could result in false positives, particularly in environments where related species may be prevalent, for example, we show up to 8% false positives in our Tanzanian cattle (PPV 0.92, Fig. 2).

Further testing is necessary to evaluate and address this potential issue. However, pairing COG with a second antigen, such as calpain, could reduce the risk of cross-reactivity, ensuring the diagnostic tool specifically detects infections with *S. bovis*. Schistosome calpains are calcium-dependent cysteine proteases found in various locations throughout the parasite’s lifecycle, including the tegument of adult worms [36, 37]. Although calpain has primarily been investigated as a vaccine candidate for the prevention and control of human and animal schistosomiasis due to its well-documented immunogenic properties [38–40], our results suggest that it also holds potential in the diagnosis of animal schistosome infections.

After validating the ELISA in our initial tests on cattle serum samples from Tanzania, we proceeded to evaluate the performance of the assay of COG and Sbp80(3) antigens using a new set of samples collected from field sites in Zanzibar in 2024. The island has a long history of human urogenital schistosomiasis caused by *S. haematobium* [41]; however, the presence of animal schistosomiasis had not been recorded until 2016, when *S. bovis*-infected *Bulinus globosus* (intermediate snail host) was identified on Zanzibar’s Pemba Island using molecular methods [42, 43]. *Schistosoma bovis* geographical overlap with *S. haematobium* in Zanzibar raises concerns about the potential for interspecies hybridization, as both parasites can infect the same intermediate host. Interspecies hybridization of schistosomes has already been detected in Zanzibar’s Unguja Island [44], as well as in other African countries, including Senegal [11] and Malawi [14]. The presence of schistosome worms in cattle was confirmed in 2019 and attributed to the livestock trade with East Africa, where *S. bovis* is more common [13].

In the present study, the animals were first evaluated for schistosomiasis by fecal egg detection using the MHT, the routine detection method for the identification of schistosomiasis of livestock in Zanzibar. MHT identified 25 out of 44 cattle as positive for schistosomiasis with a prevalence of 56.8%, presenting viable eggs that were capable of hatching within 4 min upon exposure to light. Similarly, ELISA performed with COG revealed a prevalence of bovine schistosomiasis of 59.1% (26 out of 44 cattle). Although both methods yielded comparable detection rates of *S. bovis* in the sampled cattle population in Zanzibar, one-third of the animals had opposite identification, with some testing positive by one method and negative by the other. The COG-based ELISA identified seven animals as negative for schistosomiasis that were previously positive for *S. bovis* by MHT (37% false negative, as shown by the NPV of 0.63, Fig. 5). However, all but one of these had a low level of infection according to the MHT (1–3 hatched miracidia present), suggesting that the assay may have lower sensitivity for detecting very low levels of infection.

Conversely, the COG-based ELISA also detected seven animals as positive for bovine schistosomiasis that were previously classified as negative by the MHT (28% false positive, PPV of 0.72). Two well-known limitations of MHT in regard to schistosome worms are that (i) schistosome eggs and swimming miracidia can be easily missed during the low level of infection [45] and (ii) its sensitivity is limited during the early weeks of infection, as oviposition of *S. bovis* does not commence until week 5–8 post infection [46]. In addition, eggs would not be detected after the worms are cleared. As mentioned earlier, the overlapping prevalence of *S. haematobium* and *S. bovis* is another possible explanation. Although mixed infections of *S. haematobium* and *S. bovis* in cows are considered rare, these animals could be infected with urogenital disease, which would not be detected by fecal MHT. Given the high degree of conservation exhibited by COG across the *Schistosoma* genus (Supp Fig. 1), our COG protein could still be detected by serum from an *S. haematobium*-infected animal. Clearly, additional studies are required, focusing on the interspecific specificity of these diagnostic targets.

## Conclusion

Overall, the results of our study suggest that the prevalence of bovine schistosomiasis in East Africa may be higher than previously recorded, which underscores the need for more sensitive detection methods than those currently employed. Effective control of schistosomiasis requires a One Health approach, which recognizes the interconnectedness of human, animal, and environmental health [47]. A critical aspect of this approach is the use of robust diagnostic tools for detecting animal schistosomiasis. In this concept, our leading diagnostic proteins, COG and the calpain Sbp80(3), hold a significant potential for application in East Africa, especially once developed into a portable point-of-care diagnostic test. Through facilitating the detection of infections in animal hosts, such tests are expected to enhance the surveillance efforts, inform targeted control strategies, and support the broader goal of reducing zoonotic transmission. Its application in East Africa could contribute to interrupting the transmission cycle and advancing the One Health agenda toward schistosomiasis elimination.

Following the identification of COG and the calpain Sbp80(3) as promising diagnostic targets for bovine schistosomiasis, the next phase of research needs to focus on incorporating these recombinant proteins into effective and portable point-of-care diagnostic tools, which are critically needed. While several promising point-of-care devices have recently been developed for diagnosing human schistosomiasis [48] and zoonotic *S. japonicum* infections [49, 50], there remains a noticeable gap in research and development efforts targeting animal schistosomiasis. One potential avenue for future research is to incorporate the COG and calpain into a single point-of-care device, with the aim of targeting multiple highly antigenic *S. bovis* proteins in one test. This could also help with the detection of an active infection, even at low density, and lead to enhanced diagnostic sensitivity and specificity of the test.

While our study unveils two robust *S. bovis* tegument proteins, COG and calpain, with potential as biomarkers for schistosomiasis diagnostics, this study is not without limitations. The main limitations of this study are the relatively small sample size and the opportunistic (convenience-based) sampling strategy, which together restrict the generalizability of our findings. Although we included cattle from both Tanzania and Zanzibar, detailed prevalence information from Zanzibar is lacking. Importantly, this study was not designed to estimate population-level prevalence; rather, its primary aim was to evaluate the performance of the COG- and calpain-based ELISAs. Therefore, the reported detection rates reflect only the sampled population. Further studies with larger, systematically sampled cattle populations from Zanzibar will be required to assess true prevalence and to confirm assay performance across broader settings. Additional questions remain beyond the scope of this work, including whether these proteins can distinguish between different *Schistosoma* species, detect infections in other domestic animals, or avoid cross-reactivity with other parasites.

In conclusion, our study identified two novel proteins with high diagnostic potential for *S. bovis* infection in East Africa, representing a significant advancement in the field of veterinary diagnostics. These results will be used for the development of improved diagnostic devices for livestock schistosomiasis, offering a valuable tool for more accurate epidemiological assessments and more effective interventions, aligning with global efforts to control and eliminate schistosomiasis.

## Supplementary Information

**Additional file 1:** **Table S1. **Details of NCBI BLAST search of nuclear ribosomal internal transcribed spacergene against *Schistosoma* species.

**Additional file 2:** **Table S2. **Details of the NCBI BLAST search of Oligomerix Golgicomplex subunit 4 in *Schistosoma* species

**Additional file 3:** **Figure S1**. Phylogeny analysis of Conserved Oligomerix Golgicomplex subunit 4 in *Schistosoma* species. The top BLAST protein hits were retrieved from the National Center for Biotechnology Informationdatabase and aligned using the multiple alignment using fast Fourier transformalgorithm within the NGPhylogeny.fr “One Click” pipeline. The maximum likelihood tree was inferred using PhyML with default settings. Branch lengths represent the number of nucleotide substitutions per site. The tree is presented in Newick format and visualized using the Interactive Tree of Life, EMBLtool. The primary COG protein from *S. bovis* is highlighted in red. Additional details obtained from the NCBI BLAST search are presented in the accompanying table.

## Data Availability

Data supporting the main conclusions of this study are included in the manuscript.

## Abbreviations

AUC: Area under the curve
CB: Column buffer
COG: Conserved Oligomeric Golgi complex subunit 4
%DOD Percent of Data/Dose DTT: DL-dithiothreitol
EDTA: Ethylenediaminetetraacetic acid
ELISA: Enzyme-linked immunosorbent assay
gDNA: Genomic DNA
IPP: Inorganic pyrophosphatase
IPTG: Isopropyl β-D-1-thiogalactopyranoside
LPS: Lipopolysaccharides from *Escherichia coli*
MHT: Miracidium hatching test
NPV: Negative prediction value
NTD: Neglected tropical disease
OP: Oligopeptidase
PBS-T-BSA 1%: PBS-T supplemented with 1% bovine serum albumin
PBS-T-Milk 5%: PBS-T containing 5% milk
PBS-T: 1X PBS containing 0.05% TWEEN®
PBS: Phosphate-buffered saline
PDZ: PDZ domain-containing protein
PGK: Phosphoglycerate kinase
PPV: Positive prediction value
RT: Room temperature
QPCR: Quantitative polymerase chain reaction
QUB: Queen’s University Belfast
rITS: Nuclear ribosomal internal transcribed spacer
ROC: Receiver operating characteristic
Sbp80(1–3): *S. bovis* calpain domains
STI: STI/Ubox containing protein
TMB: 3,3′,5,5′-Tetramethylbenzidine
WHO: World Health Organization
