# Supplementary material for: Improved serological testing for bovine schistosomiasis in Eastern Africa
Source: Parasit Vectors. 2026 Mar 23;19:193. doi: 10.1186/s13071-026-07332-1 (PMC13130579; doi:10.1186/s13071-026-07332-1)
Supplement: Supplementary file 1 — Additional file 1: Table S1. Details of NCBI BLAST search of nuclear ribosomal internal transcribed spacergene against Schistosoma species. [file 13071_2026_7332_MOESM1_ESM.docx]

**Supplementary Table 1:** Details of NCBI Blast search of nuclear ribosomal internal transcribed spacer (rITS) gene against *Schistosoma* species.

| **Animal #** | | **Sequenced Length (bp)** | **Query ID** | **Description** | **Scientific Name** | **Max Score** | **Total Score** | **Query Cover** | **E value** | **% Identity** | **Accession Length** | **Accession** |
| --- | --- | --- | --- | --- | --- | --- | --- | --- | --- | --- | --- | --- |
| 1 | 957 | | lcl\|Query_99917 | *Schistosoma bovis* genome assembly, chromosome: 2 | *Schistosoma bovis* | 1748 | 11278 | 0.99 | 0 | 0.999 | 50257572 | OX104095.1 |
| 2 | 952 | | lcl\|Query_60249 | *Schistosoma bovis* genome assembly, chromosome: 2 | *Schistosoma bovis* | 1748 | 11278 | 0.99 | 0 | 0.999 | 50257572 | OX104095.1 |
| 3 | 968 | | lcl\|Query_115475 | *Schistosoma bovis* genome assembly, chromosome: 2 | *Schistosoma bovis* | 1755 | 11315 | 0.98 | 0 | 0.999 | 50257572 | OX104095.1 |
| 4 | 967 | | lcl\|Query_88593 | *Schistosoma bovis* genome assembly, chromosome: 2 | *Schistosoma bovis* | 1755 | 11315 | 0.98 | 0 | 0.999 | 50257572 | OX104095.1 |
| 5 | 953 | | lcl\|Query_116765 | *Schistosoma bovis* genome assembly, chromosome: 2 | *Schistosoma bovis* | 1735 | 11213 | 0.99 | 0 | 0.997 | 50257572 | OX104095.1 |
| 6 | 77 | | lcl\|Query_119655 | *Schistosoma mansoni* | *Schistosoma mansoni* | 99 | 99 | 0.98 | 5.00E-19 | 0.908 | 494 | OQ921821.1 |
|  |  | |  | *Schistosoma sp.* | *Schistosoma* sp. | 99 | 99 | 0.98 | 5.00E-19 | 0.908 | 493 | OQ459349.1 |
| 7 | 956 | | lcl\|Query_22033 | *Schistosoma bovis* genome assembly, chromosome: 2 | *Schistosoma bovis* | 1753 | 11302 | 0.99 | 0 | 0.998 | 50257572 | OX104095.1 |
| 8 | 962 | | lcl\|Query_43869 | *Schistosoma bovis* genome assembly, chromosome: 2 | *Schistosoma bovis* | 1766 | 11368 | 0.99 | 0 | 1 | 50257572 | OX104095.1 |
| 9 | 958 | | lcl\|Query_80863 | *Schistosoma bovis* genome assembly, chromosome: 2 | *Schistosoma bovis* | 1748 | 11274 | 0.99 | 0 | 0.998 | 50257572 | OX104095.1 |
| 10 | 944 | | lcl\|Query_99669 | *Schistosoma bovis* genome assembly, chromosome: 2 | *Schistosoma bovis* | 1727 | 11176 | 0.99 | 0 | 0.999 | 50257572 | OX104095.1 |
| 11 | 952 | | lcl\|Query_22561 | *Schistosoma bovis* genome assembly, chromosome: 2 | *Schistosoma bovis* | 1738 | 11232 | 0.99 | 0 | 0.999 | 50257572 | OX104095.1 |
| 12 | 958 | | lcl\|Query_56493 | *Schistosoma bovis* genome assembly, chromosome: 2 | *Schistosoma bovis* | 1755 | 11315 | 0.99 | 0 | 0.999 | 50257572 | OX104095.1 |
| 13 | 955 | | lcl\|Query_62015 | *Schistosoma bovis* genome assembly, chromosome: 2 | *Schistosoma bovis* | 1749 | 11284 | 0.99 | 0 | 0.998 | 50257572 | OX104095.1 |
| 14 | 956 | | lcl\|Query_80095 | *Schistosoma bovis* genome assembly, chromosome: 2 | *Schistosoma bovis* | 1749 | 11284 | 0.99 | 0 | 0.998 | 50257572 | OX104095.1 |
| 15 | 953 | | lcl\|Query_124019 | *Schistosoma bovis* genome assembly, chromosome: 2 | *Schistosoma bovis* | 1738 | 11228 | 0.99 | 0 | 0.998 | 50257572 | OX104095.1 |
| 16 | 938 | | lcl\|Query_21039 | *Schistosoma bovis* genome assembly, chromosome: 2 | *Schistosoma bovis* | 1714 | 11110 | 0.99 | 0 | 1 | 50257572 | OX104095.1 |
| 17 | 948 | | lcl\|Query_124175 | *Schistosoma bovis* genome assembly, chromosome: 2 | *Schistosoma bovis* | 1744 | 11259 | 0.99 | 0 | 0.999 | 50257572 | OX104095.1 |
| 18 | 953 | | lcl\|Query_81585 | *Schistosoma bovis* genome assembly, chromosome: 2 | *Schistosoma bovis* | 1749 | 11285 | 0.99 | 0 | 0.998 | 50257572 | OX104095.1 |
| 19 | 969 | | lcl\|Query_65135 | *Schistosoma bovis* genome assembly, chromosome: 2 | *Schistosoma bovis* | 1755 | 11315 | 0.98 | 0 | 0.999 | 50257572 | OX104095.1 |
| 20 | 953 | | lcl\|Query_13891 | *Schistosoma bovis* genome assembly, chromosome: 2 | *Schistosoma bovis* | 1740 | 11241 | 0.99 | 0 | 0.999 | 50257572 | OX104095.1 |
| 21 | 959 | | lcl\|Query_17103 | *Schistosoma bovis* genome assembly, chromosome: 2 | *Schistosoma bovis* | 1749 | 11284 | 0.99 | 0 | 0.998 | 50257572 | OX104095.1 |
| 22 | 888 | | lcl\|Query_47109 | *Schistosoma bovis* internal transcribed spacer 1 | *Schistosoma bovis* | 1639 | 1639 | 0.99 | 0 | 1 | 927 | MT580958.1 |
| 23 | 960 | | lcl\|Query_127825 | *Schistosoma bovis* genome assembly, chromosome: 2 | *Schistosoma bovis* | 1751 | 11293 | 0.99 | 0 | 0.998 | 50257572 | OX104095.1 |
| 24 | 961 | | lcl\|Query_83129 | *Schistosoma bovis* genome assembly, chromosome: 2 | *Schistosoma bovis* | 1755 | 11315 | 0.99 | 0 | 0.999 | 50257572 | OX104095.1 |
| 25 | 951 | | lcl\|Query_11803 | *Schistosoma bovis* genome assembly, chromosome: 2 | *Schistosoma bovis* | 1744 | 11256 | 0.99 | 0 | 0.998 | 50257572 | OX104095.1 |
