# Supplementary figures and images for: Improved serological testing for bovine schistosomiasis in Eastern Africa
Source: Parasit Vectors. 2026 Mar 23;19:193. doi: 10.1186/s13071-026-07332-1 (PMC13130579; doi:10.1186/s13071-026-07332-1)

Tree scale: 0.01

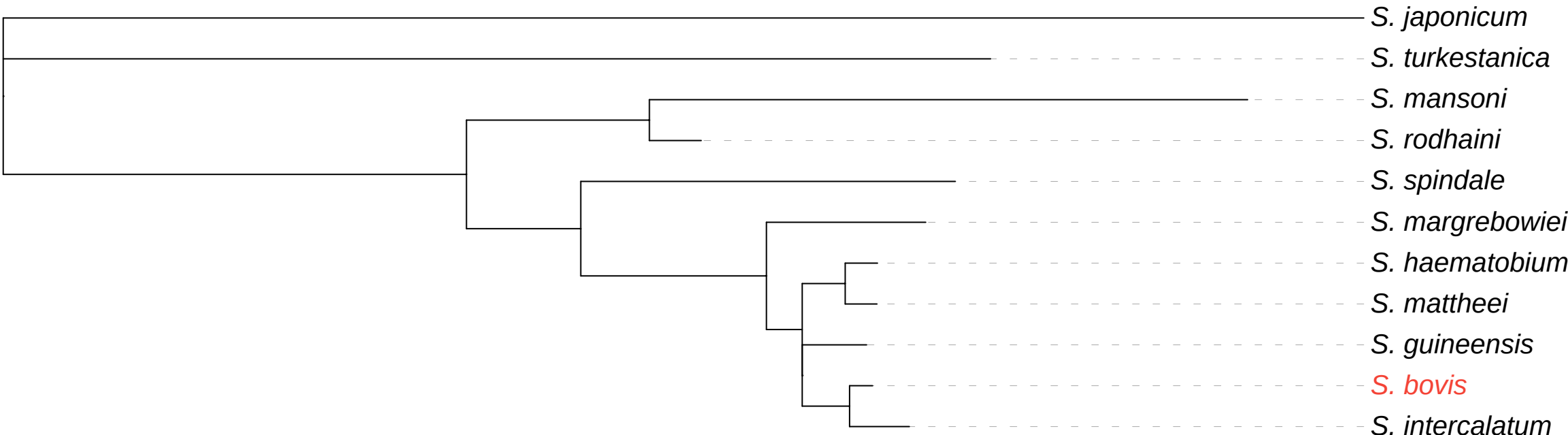

Supplement: Supplementary file 2 — Additional file 2: Table S2. Details of the NCBI BLAST search of Oligomerix Golgicomplex subunit 4 in Schistosoma species [file 13071_2026_7332_MOESM2_ESM.pdf]
