# Supplementary material for: Improved serological testing for bovine schistosomiasis in Eastern Africa
Source: Parasit Vectors. 2026 Mar 23;19:193. doi: 10.1186/s13071-026-07332-1 (PMC13130579; doi:10.1186/s13071-026-07332-1)
Supplement: Supplementary file 3 — Additional file 3: Figure S1. Phylogeny analysis of Conserved Oligomerix Golgicomplex subunit 4 in Schistosoma species. The top BLAST protein hits were retrieved from the National Center for Biotechnology Informationdatabase and aligned using the multiple alignment using fast Fourier transformalgorithm within the NGPhylogeny.fr “One Click” pipeline. The maximum likelihood tree was inferred using PhyML with default settings. Branch lengths represent the number of nucleotide substitutions per site. The tree is presented in Newick format and visualized using the Interactive Tree of Life, EMBLtool. The primary COG protein from S. bovis is highlighted in red. Additional details obtained from the NCBI BLAST search are presented in the accompanying table. [file 13071_2026_7332_MOESM3_ESM.docx]

| **Species** | **Max Score** | **Total Score** | **Query Cover** | **Percentage Identity** | **Accession Length** | **Accession** |
| --- | --- | --- | --- | --- | --- | --- |
| *Schistosoma bovis* | 1516 | 1516 | 100% | 100 | 750 | RTG82205.1 |
| *Schistosoma curassoni* | 1376 | 1376 | 100% | 88.15 | 782 | CAH8672084.1 |
| *Schistosoma mattheei* | 1364 | 1364 | 100% | 86.78 | 776 | CAH8651129.1 |
| *Schistosoma intercalatum* | 1364 | 1364 | 100% | 88.65 | 776 | CAH8657236.1 |
| *Schistosoma haematobium* | 1359 | 1359 | 100% | 86.53 | 776 | XP_035587677.2 |
| *Schistosoma guineensis* | 1355 | 1355 | 100% | 86.91 | 775 | CAH8666526.1 |
| *Schistosoma spindale* | 1276 | 1276 | 100% | 81.67 | 776 | CAI2736851.1 |
| *Schistosoma rodhaini* | 1276 | 1276 | 100% | 83.04 | 776 | CAH8646625.1 |
| *Schistosoma turkestanicum* | 1118 | 1118 | 100% | 72 | 781 | CAH8600405.1 |
| *Schistosoma japonicum* | 1112 | 1112 | 100% | 70.86 | 777 | TNN06157.1 |
| *Schistosoma mansoni* | 628 | 628 | 62% | 71.94 | 450 | XP_018644690.1 |

**Supplementary Table 2:** Details of the NCBI BLAST search of Oligomerix Golgi (COG) complex subunit 4 in *Schistosoma* species.
